# Delivering therapeutic proteins in vivo by engineered DNA-free virus-like particles

**DOI:** 10.1038/s42003-022-03338-4

**Published:** 2022-04-27

**Authors:** Theam Soon Lim

**Affiliations:** Institute for Research in Molecular Medicine, Universiti Sains Malaysia, Penang, Malaysia

## Abstract

Many genetic disorders are a result of single or multiple genome abnormalities. A possible approach to circumvent genetic disorders is to use gene editing agents to correct these mistakes, but a major challenge remains in the mode of delivery of gene editing agents to different regions of the body. Banskota et al. present the use of engineered DNA-free virus-like particles (eVLPs) to deliver base editors to different organs in a mice model for improved outcomes, highlighting the potential of eVLPS to deliver base editors and as an efficient delivery mechanism, leveraging the advantages of viral and nonviral delivery methods.

Abnormalities in the genome can bring about various genetic disorders. A possible approach for the treatment of genetic disorders is by correcting these harmful mutations. Gene editing agents have provided an avenue to manipulate genomic DNA in living organisms with great precision. This approach provides the possibility to edit the genome to alleviate the origin of many genetic diseases. A major challenge to carry out this repair is the delivery mechanism of the editing agents to different regions of the human body. Current approaches commonly use adeno-associated viruses (AAVs) to deliver the DNA that encodes for specific base editors. However, concerns surrounding the use of virus delivery systems includes prolonged expression causing off-site mutations and possible oncogenesis from viral vector integration.

Banskota et al.^1^ recently described the design of virus like particles (VLPs), which is in essence a hollow protein shell of the virus devoid of any viral genetic material, allowing for the delivery of ribonucleoproteins (RNPs) in place of DNA, to reduce the chance of off-target editing thanks to the shorter lifetime of RNPs in cells. Banskota et al. utilized these engineered VLPs (eVLPs) to deliver therapeutic RNPs, including base editors and Cas9 nuclease. The new design allows eVLPs to package 16-fold more base editor RNP compared to earlier versions of VLPs. The authors successfully applied the eVLPs for delivery to different sites in the animal host. The system was able to reduce serum Pcsk9 levels in the liver and restore visual function in a mouse model of genetic blindness.

The published system signifies the possibility of utilizing eVLPs to deliver gene editing therapies to circumvent genetic disorders at different locations in the human body with a minimized risk of off-target mutations. More importantly, the system can also be applied for the in vivo delivery of other therapeutic proteins and RNPs. The nature of the production of eVLPs means there is a possibility that cellular proteins and RNAs from the originating cell could be packaged alongside the eVLPs. Therefore, in depth protein profile analysis of the eVLPs with an optimized production system would have to be established to reduce immunogenicity issues. The pharmacokinetics of the eVLPs need to be completed to determine the half-life of eVLPS, the cargoes and dosing requirements, and although there are still some aspects of eVLPs that have yet to be clarified, the current developments bode well for the potential of eVLPs for the treatment of genetic disorders in the future.

## References

[1] Banskota S (2022). Engineered virus-like particles for efficient in vivo delivery of therapeutic proteins. Cell.

